# Rational design of a modular mRNA vaccine platform for rapid adaptation to SARS-CoV-2 variants

**DOI:** 10.1038/s41598-026-48481-8

**Published:** 2026-06-03

**Authors:** Julia Rudert, Julia Volckmar, Andreas Jeron, Maike Bälkner, Pia Grimpe, Henning Jacobsen, Dandan Zhang, Giovana Bavia Bampi, Luka Cicin-Sain, Dunja Bruder, Joseph Rosenecker, Xiaoyan Ding

**Affiliations:** 1 Department of Pediatrics, Dr. von Hauner Children’s Hospital, University Hospital, LMU Munich, Munich, Germany; 2 Immune Regulation Group, Helmholtz Centre for Infection Research, Braunschweig, Germany; 3 Infection Immunology Group, Institute of Medical Microbiology and Hospital Hygiene, Health Campus Immunology, Infectiology and Inflammation, Otto-von-Guericke University, Magdeburg, Germany; 4 Department of Viral Immunology, Helmholtz Centre for Infection Research, Braunschweig, Germany; 5 Centre for Individualized Infection medicine, Helmholtz Centre for Infection Research and the Hannover Medical School, Hannover, Germany

**Keywords:** Computational biology and bioinformatics, Immunology, Microbiology

## Abstract

The ongoing emergence of novel SARS-CoV-2 variants due to viral mutations poses a persistent challenge to the efficacy of existing vaccines. To address this challenge, we engineered and comprehensively tested three optimized mRNA vaccine candidates, evaluating the kinetics, quality, and magnitude of antibody responses as well as antigen-specific T cell immunity during a prime-boost vaccination regimen in mice. Among the tested candidates, TP2A encoding secreted receptor-binding domains (RBDs) derived from SARS-CoV-2 wild type (WT), Delta and Omicron variants demonstrated superior immunogenicity, inducing an early IgG2a-dominated antibody response against distinct SARS-CoV-2 spike (S) glycoprotein variants. In addition, TP2A elicited IFN-γ-producing T cells in both spleen and draining lymph nodes and antigen-specific cytotoxic T lymphocytes. Notably, beyond broad immunity induced by the vaccine, TP2A functions as a modular platform, thus enabling flexible antigen assembly and rapid vaccine adaptation to newly emerging variants or even other viral pathogens. These findings position TP2A as a promising next-generation mRNA vaccine candidate.

**Supplementary Information:**

The online version contains supplementary material available at 10.1038/s41598-026-48481-8.

## Introduction

Since its emergence in 2019, SARS-CoV-2 has driven the rapid development of diverse vaccine platforms, beginning with mRNA-based approaches and expanding to viral vector, protein subunit, and other technologies. Several vaccines received emergency use authorization and are now available worldwide. In the European Union, the COVID-19 vaccines currently authorized for use include Comirnaty and Spikevax (mRNA vaccines), Kostaive (self-amplifying mRNA vaccine), Bimervax and Nuvaxovid (recombinant protein subunit vaccines)^1,2^. Among these, mRNA vaccines have played a central role in global immunization efforts due to their rapid design flexibility, high immunogenicity, and scalable manufacturing^3,4^. Initially, these vaccines were constructed primarily based on the S protein of the original Wuhan-Hu-1 strain. However, as SARS-CoV-2 continued to evolve, the emergence of novel variants progressively reduced their protective efficacy^5,6^. Consequently, the ongoing viral evolution poses challenges to existing vaccines, including immune escape and waning protection^7^. Notably, mutations within the receptor-binding domain (RBD) of circulating Omicron variants have dramatically diminished vaccine efficacy^8–10^. Accordingly, several approved mRNA vaccines have been updated to better match emerging lineages. For example, the BNT162b2 (WT/BA.4/5) and mRNA-1273.222 (WT/BA.4/5) formulations incorporate sequences from Omicron subvariants to enhance protection against these prevalent strains^11,12^. Nevertheless, although scientific and manufacturing advances have enabled remarkable progress, substantial disparities in vaccine access and distribution persist worldwide - driven largely by economic, logistical, and geopolitical factors rather than technical limitations. At the same time, important scientific challenges remain partially unresolved- particularly the need to broaden and prolong vaccine-induced immunity. Together, these considerations underscore the necessity for continued research aimed at optimizing vaccine design, immunogenicity, and durability protection^13^.

The most crucial part of mRNA vaccines is the antigen they encode. The SARS-CoV-2 S protein, a type I membrane protein, forms a trimer that is anchored in the viral envelope^14^. It undergoes structural rearrangements to promote membrane fusion upon binding to the ACE2 receptor on target cells^15^. The full-length S protein consists of various domains, including the N-terminal domain (NTD), RBD, and C-terminal domains (CTD1 and CTD2) in the S1 fragment, and fusion-related domains in the S2 fragment. The NTD and in particular the RBD regions induce neutralizing antibody responses^16,17^. Licensed vaccines from Moderna and Pfizer-BioNTech use the S-2P antigen containing two proline substitutions to stabilize the prefusion conformation and thus maintaining the three-dimensional structure of the S protein, resulting in enhanced humoral immunity^18,19^. However, targeting the RBD and NTD regions of the S glycoprotein has been reported to be superior compared to S-2P mRNA vaccines^20,21^. Also, it has been shown that the HexaPro S protein structure containing six prolines (S-6P) substituting amino acids in specific positions exhibits higher stability and enhanced immunogenicity than the S-2P variant already at lower doses of the COVID-19 mRNA vaccines^22–24^.

To date, most studies have focused on improving the conformational stability of the S protein (e.g., HexaPro mutations)^23^, optimizing RBD trimer splicing strategies^25^, or inducing broadly acting immunity by tendering broad-spectrum epitopes^26^. However, these strategies do still face challenges due to the high mutation rate in certain SARS-CoV-2 strains, the occurrence of hyper-mutated variants, antigenic folding interference, decreased 3D conformational stability, and immune competition^27,28^. In order to tackle these challenges, we designed three novel mRNA vaccine candidates focusing on the S protein’s key immunodominant regions RBD and NTD. One of these candidates - the XBB-S6P construct- was designed following the established HexaPro strategy and served as an internal control. In vivo prime-boost vaccination experiments in mice revealed that the mRNA construct TP2A, which encodes RBDs from three SARS-CoV-2 variants designed to be secreted as individual proteins, elicited humoral and cellular immune responses, even outperforming the currently favored HexaPro vaccination strategy. These findings highlight TP2A´s potential as a next-generation modular mRNA vaccine candidate that can be rapidly adapted to emerging SARS-CoV-2 variants.

## Results

### Rational design of novel SARS-CoV-2 mRNA vaccine candidates

**Fig. 1:**
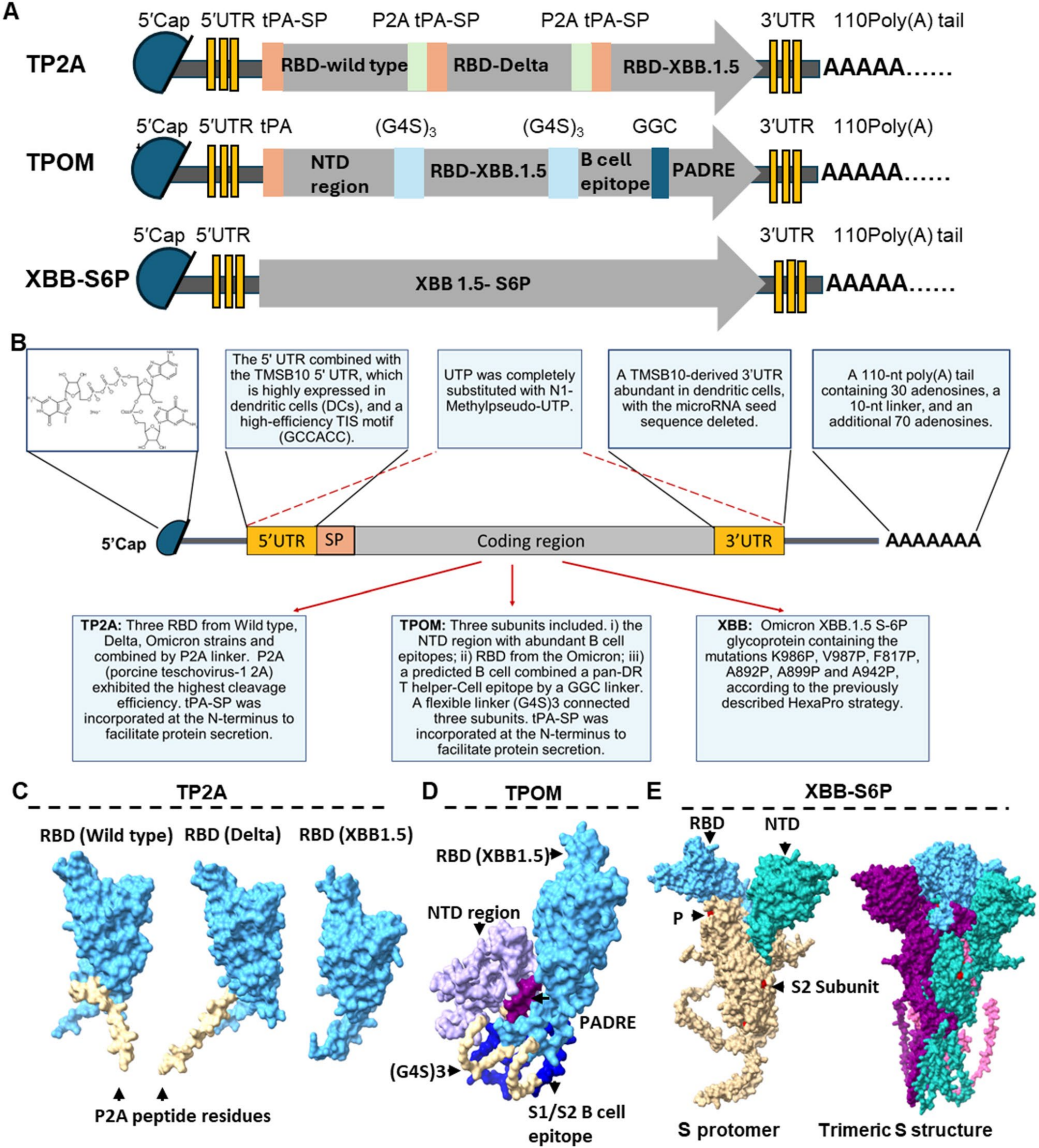
Schematic structure of the three mRNA vaccine candidates and predicted conformation of their encoded proteins. **(A)** Schematic drawing of the three SARS-CoV-2 mRNA vaccine candidates. Untranslated regions (UTRs) were incorporated upstream and downstream of the antigens of interest. All uridine residues in the mRNAs were replaced by N1-methylpseudouridine. tPA-SP: tissue plasminogen activator signal peptide; P2A: porcine teschovirus-1 2 A. **(B)** Overview of RNA design and synthesis strategy. **(C–E)** Predicted structures and conformations of the encoded proteins: **(C)** TP2A, showing three RBDs: WT (predicted template modeling (pTM) = 0.76), Delta (pTM = 0.82), and XBB1.5 (pTM = 0.76); **(D)** TPOM (pTM = 0.50); and **(E)** XBB-S6P (pTM = 0.80). Black arrows indicate relevant domains, including the RBDs, epitopes, and fusion peptides. The pTM score provides an estimate of the accuracy of the predicted structure.

To improve S protein-specific mRNA vaccine efficacy we designed three novel SARS-CoV-2 mRNA vaccine candidates containing different Open Reading Frames (ORFs) targeting different antigens, as well as optimized untranslated regions (UTRs) and poly A tails (Fig. 1A). The TP2A candidate’s ORF encodes RBDs from SARS-CoV-2 WT, Delta, and the Omicron XBB 1.5 variant connected with a P2A peptide linker that enables cleavage during translation. A human tissue plasminogen activator signal peptide (tPA-SP) coding sequence was added at the 5’ end of each RBD to facilitate protein secretion. The TPOM’s ORF consists of three subunits: (i) the NTD selected based on homologous sequence alignment of the WT and Omicron strains, showing the highest sequence similarity and being rich in B cell epitopes; (ii) the RBD from the Omicron XBB.1.5 strain; (iii) a predicted B cell epitope from the S1/S2 cleavage region and the S2 fusion subunit of S proteins, linked to a pan-DR T helper-cell epitope (PADRE, AKFVAAWTLKAAA) via a GGC linker. These sequences were linked by a flexible linker consisting of three Gly-Gly-Gly-Gly-Ser repeats (G4S)_3_. Additionally, as for TP2A, a tPA-SP coding sequence was incorporated at the N-terminus to facilitate protein secretion. The XBB-S6P´s ORF encodes the Omicron XBB.1.5 S-6P glycoprotein containing the mutations K986P, V987P, F817P, A892P, A899P and A942P, according to the previously described HexaPro strategy^23,29^. To enhance the expression of the respective mRNA encoded antigens in target cells, we selected for all three constructs UTRs that were previously reported to be predominantly expressed in antigen presenting cells^30^. Of note, we made further improvements in the 3’UTR (for sequence details, please refer to Table S1). To each mRNA vaccine candidate, an identical 110-nucleotide poly(A) tail was added. The overall concept for the mRNA design is summarized in Fig. 1B. Sequences were synthesized by GenScript as DNA template and mRNAs for TP2A, TPOM, and XBB-S6P were synthesized by co-transcription using the CleanCap AG from TriLink Biotechnologies with the N1-Me-Pseudouridine substitution method. mRNA integrity and size were evaluated by agarose gel electrophoresis. A distinct single band corresponding to the expected transcript length was observed for each construct, indicating successful transcription with minimal degradation (Supplementary Fig. 1). mRNA translation into protein was confirmed by ELISA (Supplementary Fig. 2).

To predict the protein conformation of the TP2A, TPOM, and XBB-S6P encoded antigens, we used the AlphaFold Server, an online platform powered by the latest AlphaFold 3 model, to generate de novo structure predictions^31^. P2A is known for its high ribosomal skipping efficiency, enabling the co-translational production of three separate proteins from a single ORF. This process typically leaves a short peptide remnant at the C-terminus of the upstream protein and an additional proline residue at the N-terminus of the downstream protein^32–34^. SignalP 6.0 analysis predicted that an N-terminal proline residue, resulting from P2A cleavage, does not impair signal peptide function (Supplementary Fig. 3). Therefore, TP2A translation very likely results in three individually secreted proteins, two of which (RBD-WT and RBD-Delta) retaining a residual P2A peptide sequence (GSGATNFSLLKQAGDVEENPG) at their C-terminus. Since P2A is located at the bottom of the RBD domain, it would not alter the RBD conformation according to the AlphaFold 3 predicted structure (Fig. 1C). TPOM encodes an entirely newly designed fusion protein, with the (i) and (iii) subunits predicted to be predominantly localized at the lower portion of the (ii) subunit (Fig. 1D). The protein encoded by XBB-S6P is predicted to exhibit a structure similar to that of the previously reported HexaPro protomer^23^. According to the structure prediction, the S protein protomer would be anchored to the cell membrane and spontaneously assemble into trimeric structures (Fig. 1E).

### TP2A and XBB-S6P induce humoral and cellular immune responses following prime-boost vaccination in mice

**Fig. 2:**
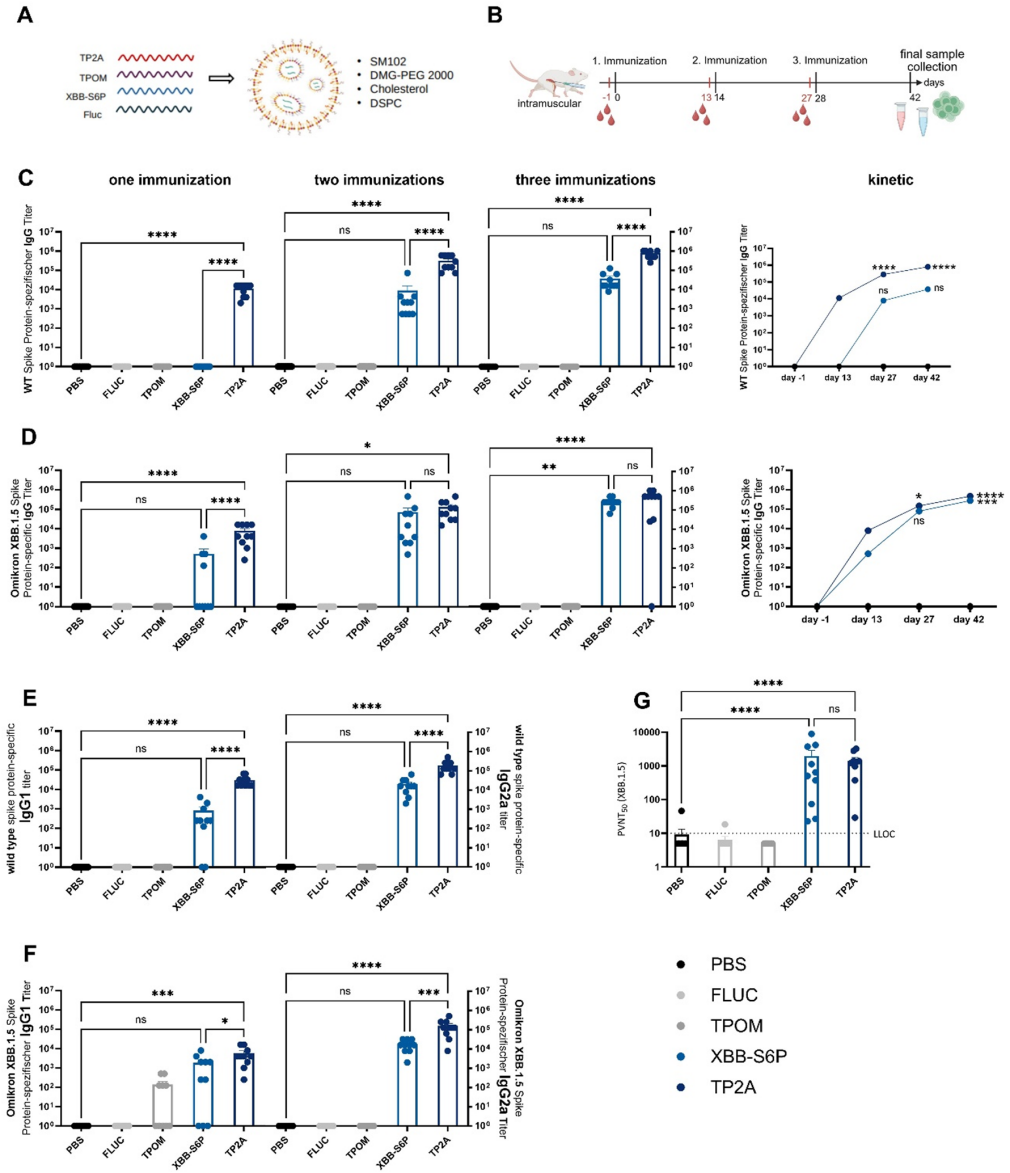
Prime-boost vaccination with TP2A and XBB-S6P, but not TPOM mRNA, induces WT and XBB.1.5 S protein-specific neutralizing antibodies. (**A**) Schematic presentation of LNP mRNA vaccine formulation. **(B)** Schematic presentation depicting immunization schedule and sample collection (Created with BioRender.com). **(C,D)** Total IgG titers against WT S or XBB.1.5 S protein. **(E,F)** Subclass analysis of IgG titers against WT S or XBB.1.5 S protein after 3 immunizations (day 42). **(G)** Pseudovirus neutralization assay. Mice were immunized with LNP-encapsulated mRNA constructs encoding TP2A, TPOM, or XBB-S6P. Sera were collected on day − 1, 13, 27 and 42 and analyzed by ELISA. S-specific total IgG, IgG1 and IgG2a levels were measured using serial dilutions against recombinant WT and XBB.1.5 S proteins. Antibodies were analyzed for their neutralizing properties using a VSV S pseudo-virus system encoding the XBB.1.5 S protein. Results represent mean ± SEM (*n* = 10 mice/group). Statistical analysis was performed using one-way ANOVA or lognormal one-way ANOVA followed by Tukey’s multiple comparisons test (* *p* < 0.05, ** *p* < 0.01, *** *p* < 0.001, **** *p* < 0.0001). (LLOC – lower limit of confidence).

To first evaluate the nature and strengths of antibody responses elicited by the newly designed mRNA vaccine constructs, BALB/c mice were immunized intramuscularly (i.m.) three times with LNP-formulated mRNAs encoding either TP2A, TPOM, or XBB-S6P on days 0, 14, and 28. As controls, mice were treated with an unrelated LNP-formulated firefly-luciferase (FLUC) mRNA or vehicle only (PBS). Serum samples were collected one day prior to each vaccination and two weeks after the final vaccination (Fig. 2A,B).

Quantification of antibody responses by ELISA revealed that in contrast to the two other candidates, already a single immunization with the TP2A vaccine elicited high WT S-specific IgG titers (Fig. 2C). While considerable WT S-specific IgG titers were obtained following the first and second booster immunization with XBB-S6P, they remained significantly lower compared to that observed after prime-boost vaccination with the TP2A mRNA (Fig. 2C). This pattern was also observed for WT RBD-specific IgG titers (Supplementary Fig. 4). Of note, TPOM failed to induce any detectable WT S- and RBD-specific IgG responses. Interestingly, the XBB.1.5 S-specific IgG response followed a similar pattern. Here, both the XBB-S6P and TP2A mRNA candidates induced an equally strong antibody response upon prime-boost vaccination, which was however delayed for the XBB-S6P mRNA vaccine (Fig. 2D). Again, as for the WT S protein, no XBB.1.5 S-protein-specific IgG response was observed following TPOM vaccination (Fig. 2D). Antibodies were also tested for their neutralizing capacities using the VSV-SARS-CoV-2 S protein pseudo-virus system. In line with ELISA data, the XBB-S6P and TP2A mRNA candidates induced equally high levels of neutralizing antibodies against the XBB 1.5 S protein, while TPOM vaccination did not (Fig. 2G).

Determination of antibody subclasses revealed prime-boost immunization with TP2A and XBB-S6P mRNAs to generally induce higher IgG2a than IgG1 titers (Fig. 2E and F). Direct comparison of the IgG2a to IgG1 titers demonstrated a highly significant increase in the WT and XBB1.5 S-specific IgG2a/IgG1 ratios for TP2A (mean endpoint titers WT S-protein: 1:192.000 (IgG2a), 1:30.400 (IgG1), IgG2a/IgG1 ratio = 6,32 (*p* < 0.0001); mean endpoint titer XBB1.5 S-protein: 1:164.000 (IgG2a), 1:9.225 (IgG1), IgG2a/IgG1 ratio = 17,78 (*p* < 0.0001)). In contrast, differences in the IgG2a to IgG1 ratio for XBB-S6P did not reach statistical significance (data not shown). This implies that especially the TP2A construct preferentially induces a Th1-dominated immune response, which is known to promote antiviral immunity.

**Fig. 3:**
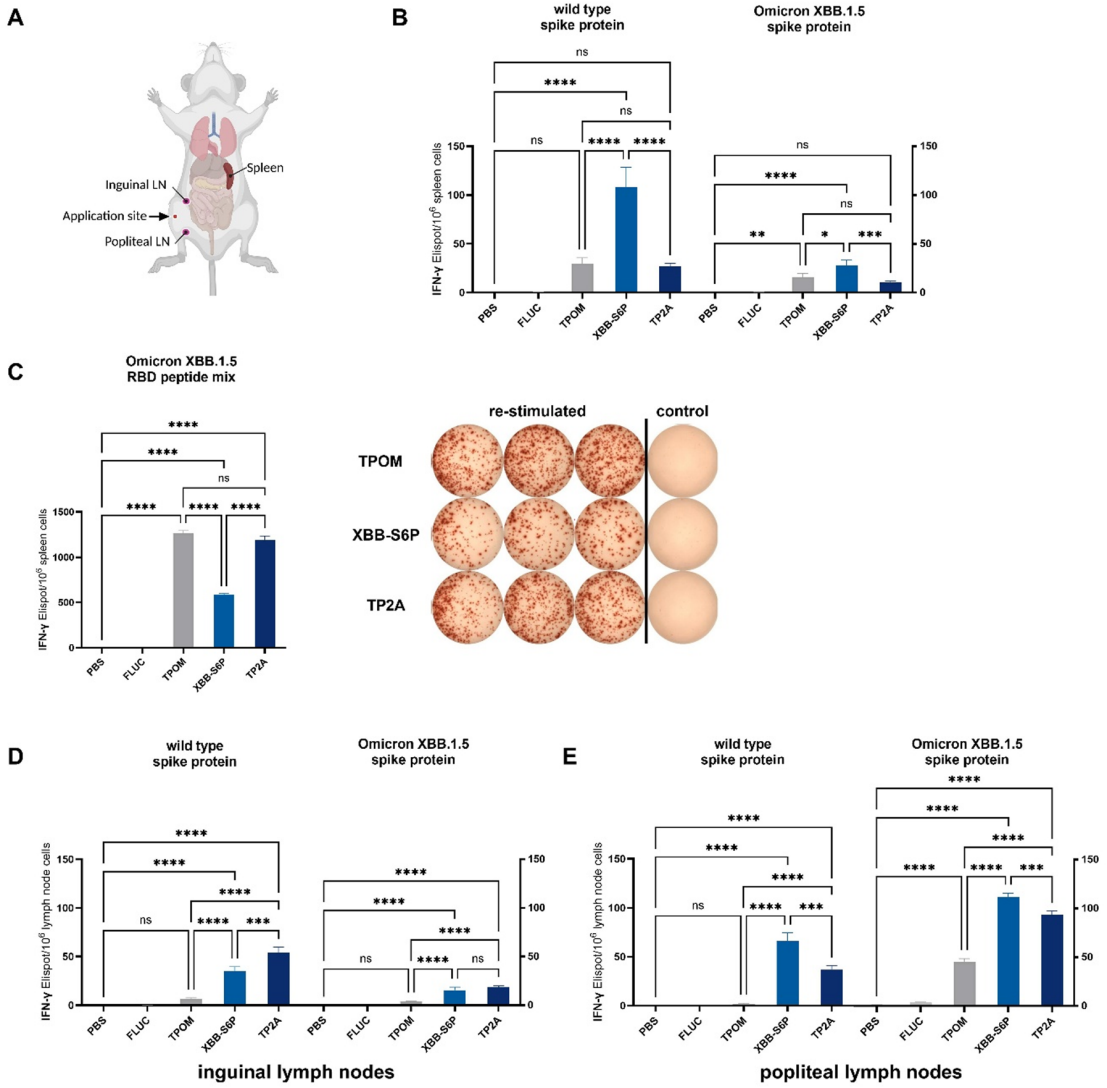
Prime-boost vaccination with TP2A and XBB-S6P mRNA induces WT and XBB.1.5 S protein-specific IFN-γ response. (**A**) Schematic illustration of immunization and sampling sites for cellular immune assays (Created with BioRender.com). **(B)** IFN-γ secreting splenocytes after stimulation with WT or Omicron XBB 1.5 S protein. **(C**) IFN-γ secreting splenocytes after stimulation with an Omicron XBB 1.5 RBD peptide mix and a representative picture of the resulting spot forming units (SFU). **(D)** IFN-γ secreting lymphocytes of the inguinal lymph nodes after stimulation with WT or Omicron XBB 1.5 S protein **(E)** IFN-γ secreting lymphocytes of the popliteal lymph nodes after stimulation with WT or Omicron XBB 1.5 S protein. Splenocytes and lymph node cells were isolated from immunized mice at day 14 after final vaccination and stimulated in vitro with recombinant WT or XBB.1.5 S proteins or XBB.1.5 RBD peptide mix. IFN-γ secretion was quantified by ELISpot. Results are presented as SFUs per 10⁶ cells. Data are expressed as means ± SEM of triplicates of pooled groups from one or two experiments (*n* = 5 or 10 mice/group). Statistical significance was determined using one-way ANOVA followed by Tukey’s multiple comparisons test (* *p* < 0.05, ** *p* < 0.01, *** *p* < 0.001, **** *p* < 0.0001).

To validate potential Th1-polarization elicited by the different mRNA candidates, IFN-γ ELISpot assays were performed using leukocytes isolated from spleen, inguinal lymph nodes (iLN) and popliteal lymph nodes (pLN). Re-stimulation was carried out using either WT or XBB.1.5 S protein or a peptide pool consisting of 53 overlapping 15-mer peptides with an 11 amino acid overlap covering the RBD of the XBB.1.5 S protein (Fig. 3). This peptide pool contained the mutations G339H, R346T, L368I, S371F, S373P, S375F, T376A, D405N, R408S, K417N, N440K, V445P, G446S, N460K, S477N, T478K, E484A, F486P, F490S, Q498R, N501Y and Y505 of the lineage B.1.1.529 (XBB.1.5 Omicron). Compared to the respective control groups, the number of IFN-γ producing splenocytes, representing a systemic proxy of vaccination-induced T cells, was significantly increased following XBB-S6P mRNA vaccination and re-stimulation with WT S protein (Fig. 3B). Similar results were obtained upon re-stimulation with the XBB.1.5 S protein, with the addition that TPOM vaccinated mice demonstrated a significant number of spot-forming units (SFU) (Fig. 3B). Re-stimulation with the XBB.1.5 S protein RBD peptide pool induced a far stronger (~ 20-fold) IFN-γ response in splenocytes for all three mRNA candidates tested, with TP2A inducing equally high numbers of IFN-γ producing cells as TPOM (Fig. 3C). Taken together, prime-boost vaccination with all three TP2A, TPOM and XBB-S6P mRNA vaccines induces a systemic Th1 cell response in mice. We further quantified the numbers of IFN-γ producing leukocytes in iLN and pLN draining the site of mRNA vaccine application. Interestingly, as for the spleen, re-stimulation with WT or XBB.1.5 S protein revealed significant numbers of IFN-γ producers following XBB-S6P immunization and, in contrast to the spleen, as well for TP2A immunization (Fig. 3D,E). However, apart from XBB.1.5 S protein re-stimulation of pLN, no IFN-γ response was detectable in TPOM immunized mice.

Taken together, the TP2A mRNA candidate induces the strongest and fastest IgG antibody response comprising both Th1-related IgG2a and Th2-related IgG1 antibody subclasses, with a significant bias towards Th1 immunity (Fig. 2). Both TP2A and XBB-S6P efficiently induce IFN-γ-producing lymphocytes. Interestingly, while compared to TP2A, the XBB-S6P construct induced a delayed humoral immune response, TPOM completely failed to induce S-protein-specific IgG (Fig. 2). This is contrasted by the presence of IFN-γ producing cells observed in splenic leukocytes from TPOM vaccinated mice, demonstrating its capacity to induce cellular immunity (Fig. 3C).

### TP2A and TPOM elicit cytotoxic CD8⁺ T cell responses

**Fig. 4:**
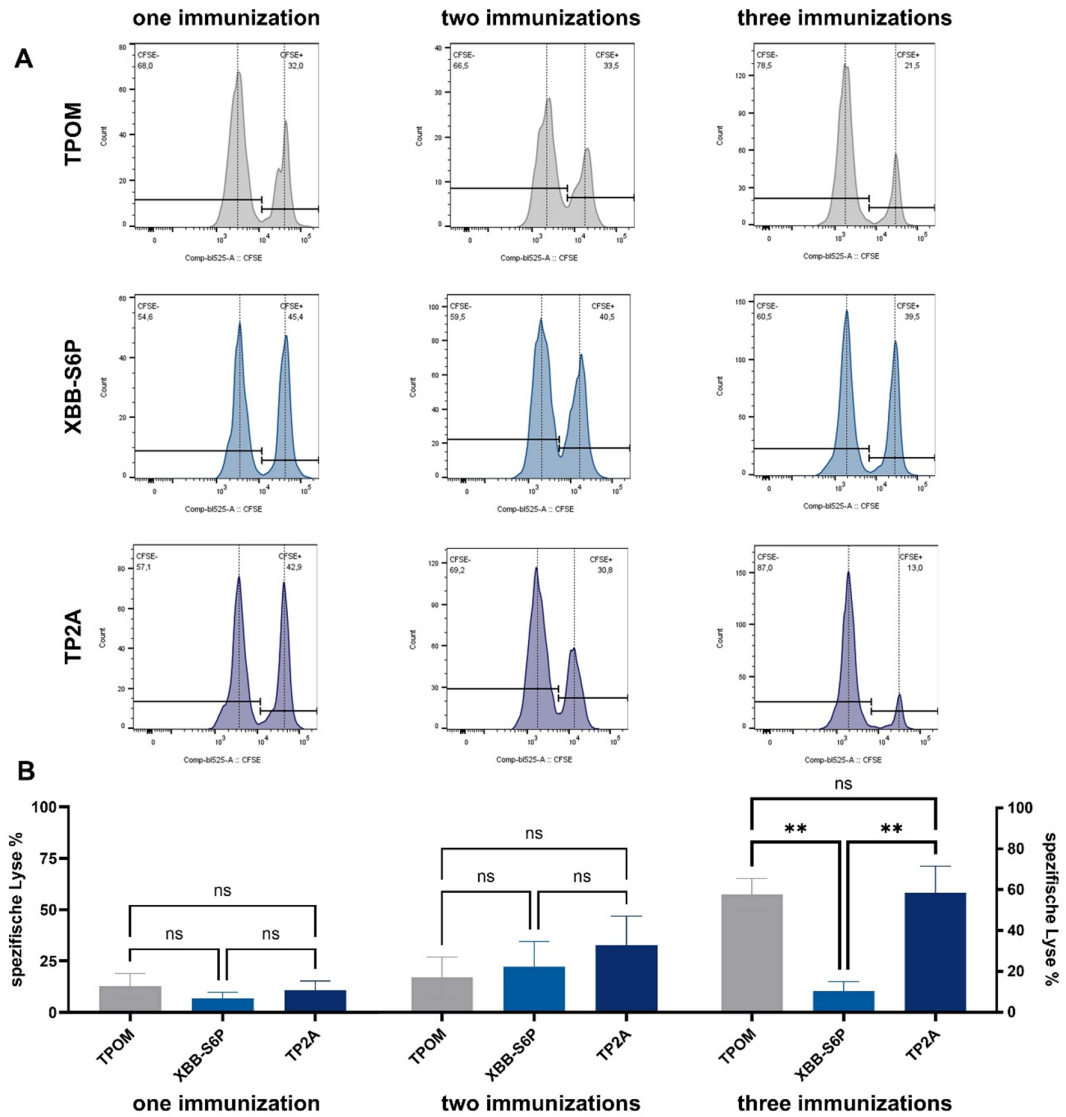
TP2A and TPOM prime-boost vaccination elicits antigen-specific cytotoxic T cells response. (**A**) Representative FACS histograms of peptide-loaded CFSE^high^ and reference CFSE^low^ cell populations. Numbers represent percent of CFSE^high/low^ cells. **(B)** Antigen-specific lysis of peptide-loaded CFSE^high^ target cells. BALB/c mice (*n* = 8 mice/group) were immunized once, twice or three times with LNP-formulated TP2A, TPOM, or XBB-S6P mRNA. Control mice received PBS. 9 days after the last immunization, CFSE-stained splenocytes pulsed with XBB.1.5 RBD-derived peptide (CGPKKSTNL) (CFSE^high^) or left untreated (CFSE^low^) were injected intravenously at a ratio of 1:1 into immunized recipient mice to determine CTL-mediated specific lysis by flow cytometry. Specific lysis of peptide-loaded CFSE^high^ cells was calculated in reference to the according CFSE^low^ population and the PBS control. Data represent mean ± SEM. Statistical significance was assessed by one-way ANOVA followed by Tukey’s multiple comparisons test (**p < 0.01).

Next, we sought to investigate the ability of each vaccine construct to induce functional antigen-specific CD8⁺ T cell responses in vivo. To this end, we tested immunized BALB/c mice for the presence of cytotoxic CD8^+^ T cells, recognizing the H-2D^d^-restricted peptide epitope CGPKKSTNL derived from the RBD of the XBB.1.5 S protein (Supplementary Fig. 5). This peptide was previously described by Muraoka and colleagues^35^. To determine, whether antigen-specific CD8⁺ T cells were induced upon vaccination and were also functionally competent, we conducted an in vivo cytotoxic T lymphocyte (CTL) assay using adoptive transfer of 5-(6)-Carboxyfluorescein succinimidyl ester (CFSE)-labeled target cells prior pulsed with the CGPKKSTNL peptide (Fig. 4A,B). Mice vaccinated three times with TP2A and TPOM exhibited a marked antigen-specific killing activity, with an average of 58.5% and 57.7% specific target cell lysis, respectively. XBB-S6P immunized mice, however, showed far less pronounced antigen-specific cytotoxicity, which did not exceed background level. Notably, the specific lysis efficiency for TPOM and TP2A increased steadily over the course of three immunizations, whereas this was not observed for the XBB-S6P mRNA candidate. These results demonstrate that prime-boost vaccination with LNP-formulated TP2A and TPOM induces the expansion of antigen-specific CD8⁺ T cells and primes them to exert effective cytolytic activity in vivo.

Collectively, our results show that among the three candidates tested the TP2A construct induces both quantitatively and functionally the strongest S protein-specific immune response which comprises the rapid induction of WT and Omicron S protein-specific antibody responses, a Th1-mediated cellular immune response favorable to antiviral immunity, and the effective induction of S protein-specific cytotoxic CD8^+^ T cells. Unlike conventional RBD fusion constructs, the TP2A design enables multiple distinct RBDs to be independently expressed and presented to the immune system, resulting in a diverse immune activation. Such broad humoral and cellular immune response is considered critical for effective viral clearance and long-term protection against divergent SARS-CoV-2 variants.

## Materials and methods

### mRNA vaccine design and synthesis

Three different recombinant SARS-CoV-2 mRNA vaccine candidates (TP2A, TPOM, and XBB-S6P) were generated, each containing distinct antigen ORFs, 5’ and 3’ untranslated regions, and a poly(A) tail. The FLUC ORF was used as a negative control (sequence provided in Table S1). The SARS-CoV-2 variants included in this study are the WT (hCoV-19/Croatia/ZG-297-20/2020, GISAID database ID: EPI_ISL_451934), Delta (B.1.617.2 lineage, GenBank: OK091006.1), and Omicron (XBB.1.5, GenBank: OQ164410.1) strains. All sequences were preceded by a T7 promoter (TAATACGACTCACTATA), inserted into the pUC57 vector, and synthesized by GenScript. mRNAs were produced using the HiScribe^®^ T7 High Yield RNA Synthesis Kit (New England BioLabs, E2040S) following the manufacturer’s protocol, with minormodifications. In each reaction, uridine was replaced with N1-methylpseudouridine (m1ψ) (Jena Bioscience, Germany). Additionally, CleanCap ^®^ AG (TriLink Biotech, USA) was added to the 20-µl reaction at a final concentration of 6 mM. After transcription, DNase I was added for 15 min, followed by purification using lithium chloride (LiCl). The RNA concentration was then measured using a Nanodrop.

### Protein structure prediction

Protein structure prediction was performed using AlphaFold Server^31^. The default parameters were used and the predicted structures were visualized and analyzed using ChimeraX 1.9.

### RNA electrophoresis

RNA samples (1 µg) were mixed with 2×RNA Gel Loading Dye (Thermo Scientific, USA), then loaded onto a 1% agarose gel and electrophoresed at 120 V for 40 min in 3-(N-morpholino) propanesulfonic acid (MOPS) buffer. RNA bands were visualized under UV illumination using an Intas GelStick Imager (Intas Science Imaging Instruments GmbH, Göttingen, Germany) to assess RNA integrity.

### Cell culture and transfection

In vitro transfection of mRNA was conducted in HEK 293 T cells using Lipofectamine^®^ 3000 reagent (Invitrogen, USA) following the manufacturer’s instructions. Briefly, HEK 293 T cells (ATCC, CRL-3216™) were cultured in DMEM (Gibco, USA) containing 10% FBS (Gibco, USA). Cell detachment was achieved using 0.25% TrypLE (Thermo Fisher Scientific, USA), and cells were seeded in 24-well plates at a density of 2.5 × 10^5^ cells in 500 µl DMEM medium containing 2% FBS. 24 h later, the cells were transfected with mRNA (0.4 µg per well) using the Lipofectamine 3000 Transfection Reagent. Supernatants were collected 24 h post transfection and stored at −80℃ until use.

### Antigen concentration determination

Antigen concentrations were determined using commercially available ELISA kits specific for the SARS-CoV-2 Delta (B.1.617.2) variant, the Omicron (B.1.1.529 sublineage BA.2) variant, and the WT SARS-CoV-2 strain. The following kits were used: SARS-CoV-2 Delta (B.1.617.2) variant Spike RBD ELISA Kit (SinoBiological, KIT40592B, China); SARS-CoV-2 (2019-nCoV) Spike RBD ELISA Kit (SinoBiological, KIT40592, China) and SARS-CoV-2 Omicron (B.1.1.529 sublineage BA.2) variant Spike RBD ELISA Kit (SinoBiological, KIT40592D, China). The experiments were conducted strictly according to the manufacturer’s instructions provided in the respective product manuals.

### Lipid nanoparticle (LNP)-encapsulation of mRNA

The mRNA-LNPs were prepared as previously described with some modifications^30^. In brief, SM-102 (MCE, 251104), DSPC (MCE, 261435), cholesterol (Sigma-Aldrich, 57–88−5), and DMG-PEG2000 (Avanti, 880151p-1 g-A-025) were dissolved in ethanol at a molar ratio of 50:10:38.5:1.5. mRNA was diluted in 50 mM citrate buffer (pH 4.0) to a concentration of 0.17 mg/mL. Lipid and mRNA solutions were mixed at an N/P ratio of 6:1 using the NanoAssemblr Ignite microfluidic platform (Precision Nanosystems) with a total flow rate of 12 mL/min and a flow rate ratio of 3:1 (aqueous phase: organic phase). The resulting LNP-formulated mRNA samples were dialyzed against PBS (pH 7.4) for 24 h using dialysis bags (Viskase, USA) and subsequently stored at 4 °C until use. The mRNA encapsulation efficiency was determined following the protocol provided with the Quant-iT RiboGreen RNA Assay Kit (Invitrogen, USA) and quantified using a microplate reader (FLUOstar Omega, BMG, Germany).

### Mice

Female BALB/cJRj mice were purchased at an age of about 8 weeks from Janvier (France) and housed under specific pathogen-free conditions according to national and institutional guidelines. All animal experiments were performed in accordance with the ARRIVE guidelines and approved by the local government agency (Niedersächsisches Landesamt für Verbraucherschutz und Lebensmittelsicherheit; file number 33.19–42502.19-04–23−00353).

### Immunization protocol

Mice were anesthetized by inhalation of isoflurane and immunized intramuscularly with LNP-formulated TP2A, TPOM, or XBB-S6P mRNA. LNP-formulated FLUC mRNA and PBS were used as controls. Each mouse was immunized with 2 µg of LNP-formulated RNA diluted in a total volume of 30 µl PBS, administered intramuscularly on days 0, 14, and 28 (Fig. 2B).

### Anesthesia and euthanasia

For immunization and blood collection on days − 1, 13 and 27, mice were shortly anesthetized by isoflurane inhalation using a small animal anesthesia system (XGI-8 Gas Anesthesia System, Caliper). Euthanasia was done by intraperitoneal (i.p.) injection of an overdose of ketamine (200 mg/kg body weight) and xylazine (20 mg/kg body weight). Death was confirmed by the onset of respiratory arrest, after which terminal cardiac blood collection was performed. This method ensures rapid loss of consciousness and deep anesthesia, thereby minimizing pain and distress prior to death.

### Sample collection and preparation

Blood samples were collected from the retro-orbital sinus on days −1, 13 and 27 and by cardiac puncture on day 42. Sera for the analysis of antigen-specific IgG and IgG1 and IgG2a subclasses were obtained by incubation of the samples for 30 min at 37 °C followed by 30 min incubation at 4 °C and subsequent centrifugation (10 min at 420 x g and 4 °C). For the isolation of lymphocytes from spleens and lymph nodes, organs from the experimental groups were pooled, transferred to PBS and passed through a 100 μm cell strainer (BD Biosciences, USA). Erythrocyte lysis was performed by osmotic shock (150 mM NH4Cl, 10 mM KHCO3, 0.1 mM EDTA (pH 7.2)) and cells were resuspended in RPMI medium supplemented with 10% v/v FCS, 1% v/v penicillin/streptomycin.

### Detection of antigen-specific total serum IgG and IgG subclasses

Antigen-specific total IgG as well as IgG1 and IgG2a subclasses were determined in serum samples by enzyme-linked immunosorbent assay (ELISA) as previously described^36^. In brief, 96-well Nunc-Immuno MaxiSorp plates (Nunc, Germany) were coated with 1 µg/ml SARS-CoV-2 (2019-nCoV) WT S protein (S1 + S2 ECD, His Tag) (Sino Biological Europe GmbH, Deutschland) or SARS-CoV-2 XBB.1.5 (Omicron) S protein (S1 + S2 ECD, His Tag) (Sino Biological Europe GmbH, Deutschland) in 0.05 M carbonate buffer (pH 9.6). After overnight incubation, differential washing steps and blocking, serial 2-fold dilutions of sera in 3% BSA/PBS were added. Antibody binding was detected using biotin-conjugated goat α-mouse IgG (Sigma, Germany), rabbit α-mouse IgG1 (Rockland Immunochemicals, USA) or rat α-mouse IgG2a (BioLegend, USA) antibodies (1 h, 37 °C), respectively, followed by streptavidin-HRPO (BD Biosciences, Germany) (30 min, 37 °C). Endpoint titers were expressed as the reciprocal value of the last serum dilution, which yielded an absorbance two times above the values of negative controls.

### Pseudo-virus neutralization assay

To prepare VSV-SARS-CoV-2 S protein pseudo-viruses, 293 T cells were transfected with pCG1 plasmids expressing the SARS-CoV-2 XBB.1.5 S protein, using calcium-phosphate. 24 h post transfection, cells were infected with a replication-deficient reporter VSV-G (VSV ∗ ΔG-Fluc) at a multiplicity of infection (MOI) of 3 for 1 h at 37 °C. Cells were washed once with PBS and medium containing anti-VSV-G antibody (culture supernatant from L1-hybridoma cells) was added to neutralize residual input virus. The cell culture supernatant was harvested after 16 h, and cellular debris was removed by centrifugation at 2000 g for 5 min at 4 °C. Aliquots were stored at − 80 °C until use. For pseudo-virus neutralization, serum samples and controls were heat-inactivated at 56 °C for 30 min. Thawed samples and controls were stored at 4 °C for no longer than 72 h, prior to use. In a 96-well microtiter plate, serum samples were two-fold serially diluted in cell culture medium (DMEM, 5% FBS, 1% P/S, 1% L-Glu) with a dilution range of 1:20 to 1:20480. Pre-diluted samples were incubated with an equal volume of SARS-CoV-2 XBB.1.5 S protein-bearing viral particles (693 [95% CI 681;705] fluorescence forming units (ffu)/well) at 37 °C for 1 h. After incubation, the sample-virus mixture was transferred to VeroE6 cells at 100% confluence which were seeded the day before. Cells were incubated at 37 °C for 24 ± 2 h, while infected cells were visualized using an IncuCyte S3 (Sartorius) performing whole-well scans (4x) in phase contrast and green fluorescence settings. Automated segmentation and counting of fluorescent foci defined as green fluorescent protein (GFP)^+^-single cells was performed using the IncuCyte GUI software (versions 2019B Rev1 and 2021B). Raw data were plotted in GraphPad prism version 9.0.2 and PVNT50 (pseudo-virus neutralization titers) was calculated with a variable slope, four-parameter regression analysis.

### ELISPOT assay

Enzyme-linked immunosorbent spot (ELISPOT) kits for the detection of murine IFN-γ (BD Biosciences, Germany) were used according to the manufacturer’s instructions. In brief, isolated lymphocytes pooled for the experimental groups were cultured in triplicates (4 × 10^6^ or 2 × 10^5^ per well) for 18 h in the presence of 10 µg/ml SARS-CoV-2 (2019-nCoV) S protein (S1 + S2 ECD, His Tag) (Sino Biological Europe GmbH, Germany) or SARS-CoV-2 XBB.1.5 (Omicron) S protein (S1 + S2 ECD, His Tag) (Sino Biological Europe GmbH, Deutschland) or 5 µg/ml PepMix™ SARS-CoV-2 (S-RBD XBB.1.5) (PT Peptide Technologies GmbH, Deutschland). IFN-γ-positive spots were counted using an ImmunoSpot^®^ Analyzer (Cellular Technology Limited, USA) and were analyzed using the CTL Switchboard 2.7.2 software 2.7.2 (Cellular Technology Limited, USA).

### In vivo cytotoxic T cell assay

Spleen and lymph node (mandibular, inguinal) cells were isolated from naive donor BALB/cJRj mice. Equal cell numbers were pulsed with 1 µg/ml CGPKKSTNL peptide (Thermo Fisher Scientific, custom peptide synthesis) in serum-free Iscove’s Modified Dulbecco’s Medium (IMDM, Thermo Fisher, USA) for 30 min at 37 °C or were left untreated. Following staining with the CFSE Cell Division Tracker Kit (Biolegend, USA) for 20 min at 37 °C with either 3 µM CFSE of the peptide-loaded fraction (CFSE^high^) or 0.3 µM CFSE of the non-loaded cells (CFSE^low^), the CFSE^high^ and CFSE^low^ cell fractions were mixed in a 1:1 cell-to-cell ratio. A total of 2 × 10^7^ target cells was injected intravenously into previously vaccinated or PBS control BALB/cJRj mice. After 16 h, the splenocytes of recipient mice were isolated and analyzed by flow cytometry. The percentages of CFSE^high^ and CFSE^low^ cells in reference to the total CFSE-population (CFSE^high^ + CFSE^low^) was determined, respectively. The ratio r [r = % CFSE^low^/% CFSE^high^] and the specific cell lysis L [L = 100 × (1 - (r_control_/r_vaccinated_)] was calculated.

### Data analysis

All statistical analyses were conducted using the GraphPad Prism V9.0 software. The ANOVA-One way was employed for statistical comparisons between groups.

## Discussion

In this study, three innovative mRNA vaccine constructs were designed and comprehensively characterized in vivo regarding their capacity to induce humoral and cellular immune responses against SARS-CoV-2 S-proteins. Although the TPOM construct integrated multiple common B-cell epitope regions, experimental data showed that it failed to induce detectable humoral immune responses against the SARS-CoV-2 S-protein after immunization. This result suggests that the conformational organization of TPOM may not be conducive to the spatial exposure or conformational maintenance of key RBD epitopes of the S-protein. The RBD structure contains multiple highly conformationally sensitive neutralizing epitopes such as the receptor binding module (RBM), and their correct three-dimensional presentation is crucial for B-cell recognition and the generation of neutralizing antibodies^37,38^. If spatial interference or structural folding topology changes between the multi-modal modules of TPOM, it may mask these key epitopes or alter their natural conformation, thereby affecting B-cell recognition and the formation of humoral immune responses. An alternative explanation for the observed absence humoral immunity might be that the TPOM encoded protein is not efficiently released from its producing cells. Although the TPOM construct failed to induce significant humoral immunity against both, S- (Fig. 2) and RBD-proteins (Supplementary Fig. 4), enhanced T cell responses were observed (Figs. 3 and 4), indicating efficient translation of the TPOM mRNA into protein. This phenomenon suggests that the TPOM is more suitable in its design or structural organization for subsequent antigen processing and MHC class I/II antigen presentation resulting in efficient CD4⁺ and CD8⁺ T cell activation.

The XBB-S6P vaccine candidate was designed and synthesized based on the HexaPro strategy. Consistent with previous reports^39^, this vaccine design can elicit strong humoral and cellular immune responses, especially showing significant immune efficacy against the Omicron variant. However, it failed to induce cytotoxic T cells (Fig. 3B), which can be considered a significant drawback in terms of conferring sterilizing immunity.

Compared with the other two candidates, the TP2A mRNA vaccine incorporating the P2A segmentation strategy showed optimal performance in both inducing humoral and cellular immune responses. Our data show a high IgG2a/IgG1 ratio induced by TP2A vaccination, suggesting a Th1-dominated humoral immune response (Fig. 2E, F). ELISpot data also demonstrate the generation of antigen-specific IFN-γ-secreting lymphocytes (Fig. 3), further corroborating a TP2A-induced Th1 response, which is in favor of supporting an antiviral mode of action. Although the S protein-specific IFN-γ response induced by TP2A was somewhat weaker compared to that induced by XBB-S6P vaccination, TP2A very effectively promoted the expansion of antigen-specific cytotoxic CD8⁺ T cells (Fig. 4B). These findings indicate a functional advantage that favors TP2A over XBB-S6P as a vaccine candidate capable of inducing a broader spectrum of humoral and cellular immune responses.

The efficacy of TP2A is attributed to several innovative features. First, the TP2A construct utilizes the self-shearing mechanism of P2A peptide to generate three independent SARS-CoV-2 RBD proteins during mRNA translation^33,40,41^. Despite the P2A peptides retained at the C-terminus of the RBD, our computational predictions revealed that these residues are unlikely to disrupt either the spatial conformation or the antigenicity of the RBD. In agreement with this notion, TP2A efficiently induced immune responses in vivo. Second, we added tPA-SP upstream to each RBD to improve release of antigen to the extracellular space. Previous mRNA vaccine strategies, including bivalent and trivalent chimeric S constructs^42–44^ have demonstrated the feasibility of combining S components from multiple coronaviruses within a single mRNA formulation. The RBD trimeric antigenic structure is also widely used in the current literature^45–47^. While these strategies although enhanced immunogenicity, are structurally prone to misfolding, aggregation^48,49^ or epitope masking problems^50^, limiting its broad applicability.

Furthermore, other multivalent mRNA vaccine strategies, such as BNT162b2 (WT/BA.4/5)^11^ and PTX-COVID19-M1.2^51^, rely on co-encapsulation of multiple separate mRNAs within a single LNP formulation. Although this design allows flexible incorporation of different variant antigens, it introduces potential challenges such as uneven encapsulation efficiency, differential translation, and increased manufacturing complexity.

In contrast, the TP2A construct encodes three variant RBDs within a single mRNA sequence separated by P2A peptides. This design ensures coordinated antigen expression, reduces formulation complexity, and may facilitate rapid adaptation to emerging variants. While this compact approach avoids challenges related to variable encapsulation efficiency and translation competition among separate mRNAs, it is limited by mRNA length constraints and requires further validation to confirm its efficacy compared to existing bivalent or multivalent vaccines.

Previous reports clearly indicate that Th1-type immune responses and activation of multifunctional T cells play a critical role in defense against SARS-CoV-2 infection and cannot be ignored in vaccine-induced long-term protection^52,53^. Therefore, we believe that TP2A has the potential to be used as a next-generation mRNA vaccine candidate. However, the broad-spectrum protective effects of TP2A against SARS-CoV-2 infection remain unexplored in this study and deserve further investigations. These should include (1) viral neutralization experiments to clarify its functionality for humoral immunity, (2) validation in non-human primate models to assess its protective efficacy and safety, and (3) cross-reactivity analyses against different variants to confirm its broad-spectrum protective potential.

In summary, we designed a novel mRNA vaccine that performed well in inducing both humoral and cellular immunity, providing a valuable experimental basis for the iterative upgrading and multi-variant adaptation. As TP2A features RBDs of multiple widely spread SARS-CoV-2 variants, it is designed to have cross-immunization potential and can be expected to serve as a “modular platform” to deal with emerging variants (e.g., JN.1 or CH.1.1, etc.) in the future. This modular expression framework may be broadly applicable to other RNA viruses characterized by high genetic variability, e.g. influenza A and dengue virus.

## Supplementary Information

Below is the link to the electronic supplementary material.

Supplementary Material 1

Supplementary Material 2

## Data Availability

The authors declare that the data supporting the findings of this study are available within this article and its supplementary information. [Source data](.) are provided with this manuscript. The vaccine candidates evaluated in this study were selected based on an internal preliminary screening experiment. The corresponding screening data are available from the corresponding author upon reasonable request.

## References

[1] Pattyn, J. et al. Overview of vaccines for adults authorized, recommended, and implemented in the European Union. *NPJ Vacc.* **10**, 183 (2025). 10.1038/s41541-025-01242-6 PMC12322272 40760062

[2] COVID-19 medicines | European Medicines Agency (EMA).https://www.ema.europa.eu/en/human-regulatory-overview/public-health-threats/coronavirus-disease-covid-19/covid-19-medicines(2023).

[3] Pardi, N. & Krammer, F. mRNA vaccines for infectious diseases — advances, challenges and opportunities. *Nat. Rev. Drug Discov.* **23**, 838–861 (2024). 39367276 10.1038/s41573-024-01042-y PMC13101425

[4] Zhang, Z. et al. Research progress of mRNA vaccines for infectious diseases. *Eur. J. Med. Res.* **30**, 792 (2025). 40847375 10.1186/s40001-025-03060-x PMC12374293

[5] Wack, S., Patton, T. & Ferris, L. K. COVID-19 vaccine safety and efficacy in patients with immune-mediated inflammatory disease: Review of available evidence. *J. Am. Acad. Dermatol.* **85**, 1274–1284 (2021). 34363909 10.1016/j.jaad.2021.07.054 PMC8336973

[6] Smith, T. R. F. et al. Immunogenicity of a DNA vaccine candidate for COVID-19. *Nat. Commun.* **11**, 2601 (2020). 32433465 10.1038/s41467-020-16505-0 PMC7239918

[7] Batool, S., Chokkakula, S., Jeong, J. H., Baek, Y. H. & Song, M. S. SARS-CoV-2 drug resistance and therapeutic approaches. *Heliyon* **11**, e41980 (2025). 39897928 10.1016/j.heliyon.2025.e41980 PMC11786845

[8] Willett, J.B. et al. SARS-CoV-2 Omicron is an immune escape variant with an altered cell entry pathway. *Nat. Microbiol.* **7**, 1161–1179 (2022). 35798890 10.1038/s41564-022-01143-7 PMC9352574

[9] Shah, M. & Woo, H. G. Omicron: A heavily mutated SARS-CoV-2 variant exhibits stronger binding to ACE2 and potently escapes approved COVID-19 therapeutic antibodies. *Front. Immunol.* **12**, 830527 (2022). 35140714 10.3389/fimmu.2021.830527 PMC8819067

[10] Rajsri, K. S., Singh, M. & Rao, M. Efficacy of COVID-19 vaccines against the Omicron variant of SARS-CoV-2: A review. *Explor. Res. Hypothesis Med.* **9**, 128–137 (2024).

[11] Usdan, L. et al. A bivalent Omicron-BA.4/BA.5-adapted BNT162b2 booster in ≥ 12-year-olds. *Clin. Infect. Dis.* **78**(5), 1194–1203 (2024). 38016021 10.1093/cid/ciad718 PMC11093671

[12] Chalkias, S. et al. Original SARS-CoV-2 monovalent and Omicron BA.4/BA.5 bivalent COVID-19 mRNA vaccines: Phase 2/3 trial interim results. *Nat. Med.* **29**, 2325–2333 (2023). 37653342 10.1038/s41591-023-02517-y PMC10504066

[13] Shukla,D. D. & Vora, D. K. Regulatory and global challenges in the approval, accessibility, and monitoring of mRNA vaccines post COVID-19: A review. *J. Regul. Sci.* **13**, (2025).

[14] Abedi, F., Rezaee, R., Hayes, A., Wallace, N. & Somayyeh Karimi, G. MicroRNAs and SARS-CoV-2 life cycle, pathogenesis, and mutations: biomarkers or therapeutic agents?. *Cell Cycle* **20**, 143–153 (2021). 33382348 10.1080/15384101.2020.1867792 PMC7889196

[15] Hoffmann, M. et al. SARS-CoV-2 cell entry depends on ACE2 and TMPRSS2 and is blocked by a clinically proven protease inhibitor. *Cell* **181**, 271-280.e8 (2020). 32142651 10.1016/j.cell.2020.02.052 PMC7102627

[16] Chi, X. et al. A neutralizing human antibody binds to the N-terminal domain of the Spike protein of SARS-CoV-2. *Science* **369**, 650–655 (2020). 32571838 10.1126/science.abc6952 PMC7319273

[17] Yang, J. et al. A vaccine targeting the RBD of the S protein of SARS-CoV-2 induces protective immunity. *Nature* **586**, 572–577 (2020). 32726802 10.1038/s41586-020-2599-8

[18] Xia, X. Domains and functions of spike protein in SARS-Cov-2 in the context of vaccine design. *Viruses* **13**, 109 (2021). 33466921 10.3390/v13010109 PMC7829931

[19] Noor, R. Developmental status of the potential vaccines for the mitigation of the COVID-19 pandemic and a focus on the effectiveness of the Pfizer-BioNTech and Moderna mRNA vaccines. *Curr. Clin. Microbiol. Rep.* **8**, 178–185 (2021). 33686365 10.1007/s40588-021-00162-y PMC7927780

[20] Kleanthous, H. et al. Scientific rationale for developing potent RBD-based vaccines targeting COVID-19. *npj Vaccines* **6**, 128 (2021). 10.1038/s41541-021-00393-6 PMC8553742 34711846

[21] Tian, S. et al. Distinct BCR repertoires elicited by SARS-CoV-2 RBD and S vaccinations in mice. *Cell Discov* **7**, 91(2021). 10.1038/s41421-021-00331-9 PMC8495183 34620836

[22] Wang, M.-Y. et al. SARS-CoV-2: Structure, biology, and structure-based therapeutics development. *Front Cell. Infect. Microbiol* **10**, (2020). 10.3389/fcimb.2020.587269 PMC7723891 33324574

[23] Hsieh, C.-L. et al. Structure-based design of prefusion-stabilized SARS-CoV-2 spikes. Science **369**, 1501–1505 (2020). 10.1126/science.abd0826 PMC7402631 32703906

[24] Zhu, Z. et al. S–6P exhibits better immunogenicity than S–2P at lower doses of COVID-19 mRNA vaccines. *Decod. Infect. Transm.* **2**, 100017 (2024).

[25] Juraszek, J. et al. Stabilizing the closed SARS-CoV-2 spike trimer. *Nat. Commun.* **12**, 244 (2021). 33431842 10.1038/s41467-020-20321-x PMC7801441

[26] Martin, W. R. & Cheng, F. A rational design of a multi-epitope vaccine against SARS-CoV-2 which accounts for the glycan shield of the spike glycoprotein. *J. Biomol. Struct. Dyn.* **40**, 7099–7113 (2022). 33715598 10.1080/07391102.2021.1894986 PMC9003619

[27] Harvey, W. T. et al. SARS-CoV-2 variants, spike mutations and immune escape. *Nat. Rev. Microbiol.* **19**, 409–424 (2021). 34075212 10.1038/s41579-021-00573-0 PMC8167834

[28] Abduljaleel, Z. Decoding SARS-CoV-2 variants: Mutations, viral stability, and breakthroughs in vaccines and therapies. *Biophys. Chem.* **320–321**, 107413 (2025). 39987705 10.1016/j.bpc.2025.107413

[29] Lu, M. et al. SARS-CoV-2 prefusion spike protein stabilized by six rather than two prolines is more potent for inducing antibodies that neutralize viral variants of concern. *Proc. Natl. Acad. Sci. U. S. A.* **119**, e2110105119 (2022). 35994646 10.1073/pnas.2110105119 PMC9436349

[30] Ding, X., Zhou, Y., He, J., Zhao, J. & Li, J. Enhancement of SARS-CoV-2 mRNA vaccine efficacy through the application of TMSB10 UTR for superior antigen presentation and immune activation.. *Vaccines* **12**, 432 (2024). 38675814 10.3390/vaccines12040432 PMC11053782

[31] Abramson, J. et al. Accurate structure prediction of biomolecular interactions with AlphaFold 3. *Nature* **630**, 493–500 (2024). 38718835 10.1038/s41586-024-07487-w PMC11168924

[32] Sharma, P. et al. 2A peptides provide distinct solutions to driving stop-carry on translational recoding. *Nucleic Acids Res.* **40**, 3143–3151 (2012). 22140113 10.1093/nar/gkr1176 PMC3326317

[33] Kim, J. H. et al. High cleavage efficiency of a 2A peptide derived from Porcine Teschovirus-1 in human cell lines, zebrafish and mice.. *PLoS One* **6**, e18556 (2011). 21602908 10.1371/journal.pone.0018556 PMC3084703

[34] Fang, J. et al. Stable antibody expression at therapeutic levels using the 2A peptide. *Nat. Biotechnol.* **23**, 584–590 (2005). 15834403 10.1038/nbt1087

[35] Muraoka, D. et al. Identification of a dominant CD8 + CTL epitope in the SARS-associated coronavirus 2 spike protein.. *Vaccine* **38**, 7697–7701 (2020). 33164796 10.1016/j.vaccine.2020.10.039 PMC7571903

[36] Volckmar, J. et al. Targeted antigen delivery to dendritic cells elicits robust antiviral T cell-mediated immunity in the liver. *Sci. Rep.* **7**, 43985 (2017). 28266658 10.1038/srep43985 PMC5339819

[37] He, Y., Lu, H., Siddiqui, P., Zhou, Y. & Jiang, S. Receptor-binding domain of Severe Acute Respiratory Syndrome Coronavirus spike protein contains multiple conformation-dependent epitopes that induce highly potent neutralizing antibodies. *J. Immunol.* **174**, 4908–4915 (2005). 15814718 10.4049/jimmunol.174.8.4908

[38] Huang, Q., Han, X. & Yan, J. Structure-based neutralizing mechanisms for SARS-CoV-2 antibodies.. *Emerg. Microbes Infect.* **11**, 2412–2422 (2022). 36106670 10.1080/22221751.2022.2125348 PMC9553185

[39] Bong, Y.-S. et al. S6P mutation in Delta and Omicron variant spike protein significantly enhances the efficacy of mRNA COVID-19 vaccines.. *Front. Immunol.* **15**, 1495561 (2025). 39830514 10.3389/fimmu.2024.1495561 PMC11739128

[40] Radcliffe, P. A. & Mitrophanous, K. A. Multiple gene products from a single vector: ‘Self-cleaving’ 2A peptides. *Gene Ther.* **11**, 1673–1674 (2004).

[41] de Felipe, P. et al. *Eunum pluribus*: multiple proteins from a self-processing polyprotein. *Trends Biotechnol.* **24**,68–75 (2006). 10.1016/j.tibtech.2005.12.006 16380176

[42] Wang, J. et al. Trivalent mRNA vaccine against SARS-CoV-2 and variants with effective immunization. *Mol. Pharmaceutics* **20**, 4971–4983 (2023). 10.1021/acs.molpharmaceut.2c00860 37699256

[43] Martinez, D. R. et al. Chimeric spike mRNA vaccines protect against Sarbecovirus challenge in mice. *Science* **373**, 991–998 (2021). 34214046 10.1126/science.abi4506 PMC8899822

[44] Hao, T. et al. A chimeric mRNA vaccine of S-RBD with HA conferring broad protection against influenza and COVID-19 variants. *PLoS Pathog.* **20**, e1012508 (2024). 39303003 10.1371/journal.ppat.1012508 PMC11414905

[45] Zhang, Y. et al. Broad protective RBD heterotrimer vaccines neutralize SARS-CoV-2 including Omicron sub-variants XBB/BQ.1.1/BF.7. *PLoS Pathog.* **19**, e1011659 (2023). 37721934 10.1371/journal.ppat.1011659 PMC10538664

[46] He, C. et al. A self-assembled trimeric protein vaccine induces protective immunity against Omicron variant. *Nat. Commun.* **13**, 5459 (2022). 36115859 10.1038/s41467-022-33209-9 PMC9482656

[47] He, C. et al. Trimeric protein vaccine based on Beta variant elicits robust immune response against BA.4/5-included SARS-CoV-2 Omicron variants. *Mol. Biomed.* **4**, 9 (2023). 36894743 10.1186/s43556-023-00121-7 PMC9998262

[48] Klausberger, M. et al. Designed SARS-CoV-2 receptor binding domain variants form stable monomers. *Biotechnol. J.* **17**, 2100422 (2022). 35078277 10.1002/biot.202100422 PMC9011732

[49] Moro-Pérez, L. et al. Conformational characterization of the mammalian-expressed SARS-CoV-2 recombinant receptor binding domain, a COVID-19 vaccine. *Biol. Res.* **56**, 22 (2023). 37150832 10.1186/s40659-023-00434-5 PMC10164616

[50] Lin, W.-S., Chen, I.-C., Chen, H.-C., Lee, Y.-C. & Wu, S.-C. Glycan masking of epitopes in the NTD and RBD of the spike protein elicits broadly neutralizing antibodies against SARS-CoV-2 variants. *Front. Immunol.* **12**, 795741 (2021). 10.3389/fimmu.2021.795741 PMC8674692 34925381

[51] Liu, J. et al. A bivalent COVID-19 mRNA vaccine elicited broad immune responses and protection against Omicron subvariants infection. *Npj Vacc.* **10**, 4 (2025). 10.1038/s41541-025-01062-8 PMC11718203 39788981

[52] Painter, M. M. et al. Rapid induction of antigen-specific CD4 + T cells is associated with coordinated humoral and cellular immunity to SARS-CoV-2 mRNA vaccination. *Immunity* **54**, 2133–2142 (2021). 34453880 10.1016/j.immuni.2021.08.001 PMC8361141

[53] Scaglione, A. et al. Combination of a Sindbis-SARS-CoV-2 spike vaccine and αOX40 antibody elicits protective immunity against SARS-CoV-2 induced disease and potentiates long-term SARS-CoV-2-specific humoral and T-cell immunity. *Front. Immunol.* **12**, 719077 (2021). 34394127 10.3389/fimmu.2021.719077 PMC8359677

