# Supplementary material for: Rational design of a modular mRNA vaccine platform for rapid adaptation to SARS-CoV-2 variants
Source: Sci Rep. 2026 Jun 3;16:17178. doi: 10.1038/s41598-026-48481-8 (PMC13234338; doi:10.1038/s41598-026-48481-8)
Supplement: Supplementary file 2 — Supplementary Material 2 [file 41598_2026_48481_MOESM2_ESM.pdf]

**Supplementary information**

**A** **B**

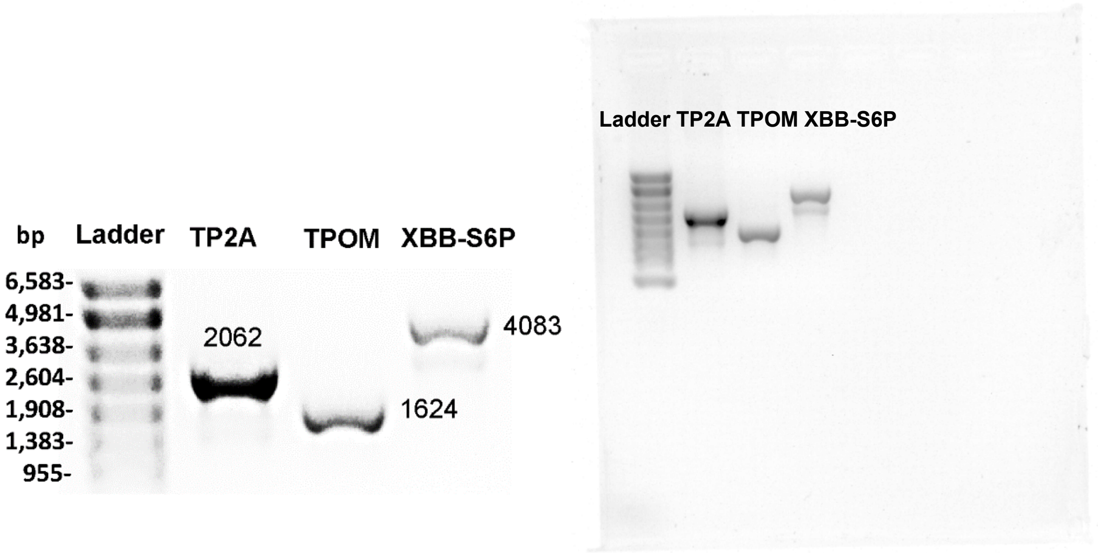


**Supplementary Figure 1:** **(A)** Agarose gel electrophoresis of *in vitro* transcribed RNA. RNA samples were transcribed *in vitro* and purified using lithium chloride precipitation. Lane 1: RNA ladder; Lane 2 – 4: *in vitro* transcribed RNA samples. The RNA was separated on a 1 % denaturing agarose gel. **(B)** The original uncropped gel.

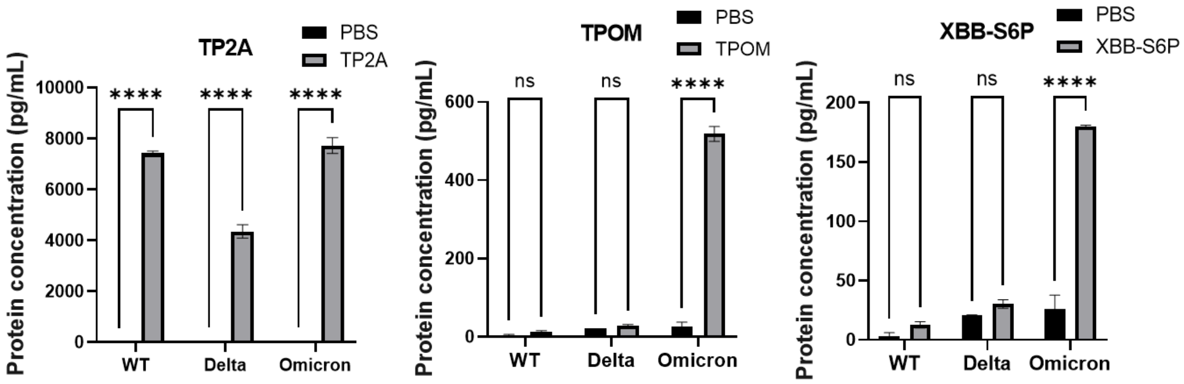


**Supplementary Figure 2:** The protein concentration of different vaccine candidates after transfected into 293T cells. Results are presented as mean ± SEM (n = 3). Adjusted P values were determined by a two-way ANOVA followed by Tukey's multiple comparisons test. ****$p < 0.0001$.

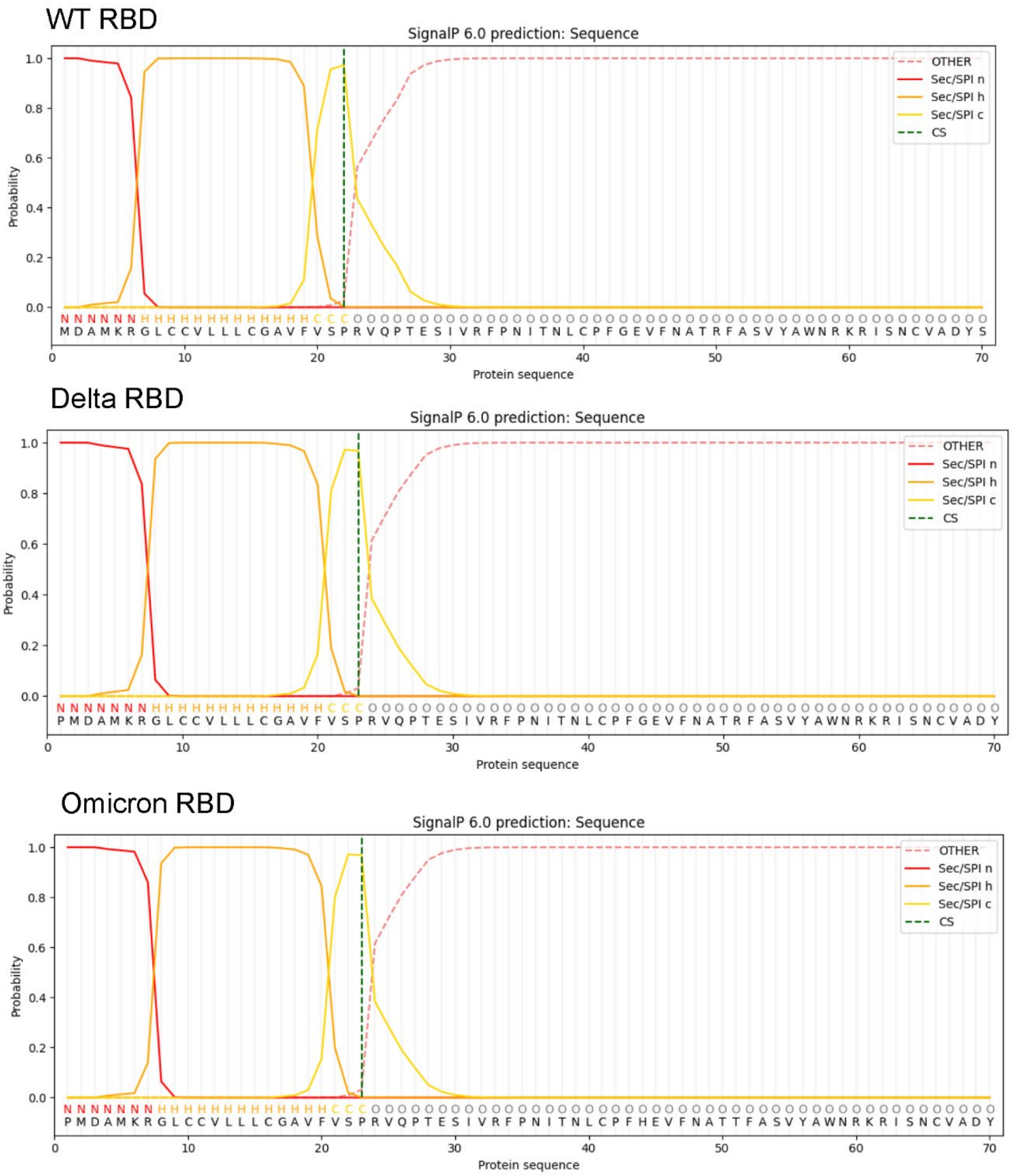


**Supplementary Figure 3**: The additional proline residue (P) that left at the N-terminus due to P2A has no effect on signal peptide activity predicted by SignalP-6.0.

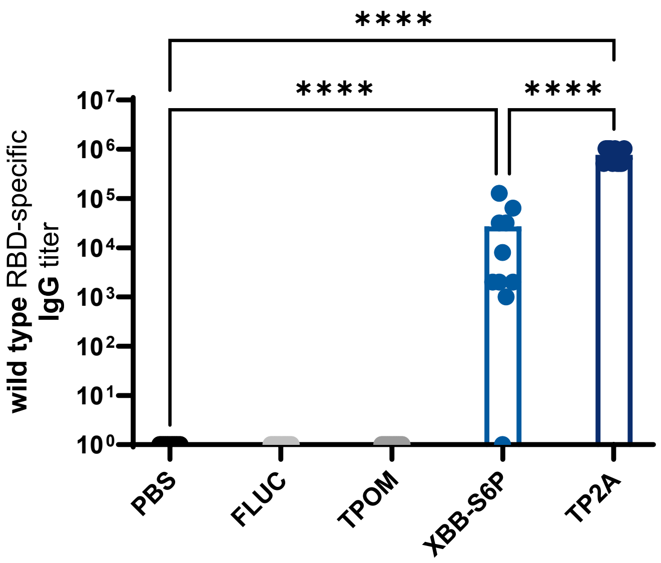


**Supplementary Figure 4:** Total IgG titers specific for the WT receptor binding domain (RBD) protein. Mice were immunized three times in a two-week interval with LNP-encapsulated mRNA constructs encoding TP2A, TPOM, or XBB-S6P. Sera were collected on day 42 and analyzed by ELISA. RBD-specific total IgG levels were measured using serial dilutions against recombinant WT RBD proteins. Results represent mean ± SEM (n = 10 mice/group). Statistical analysis was performed using lognormal one-way ANOVA followed by Tukey's multiple comparisons test (**** $p < 0.0001$).

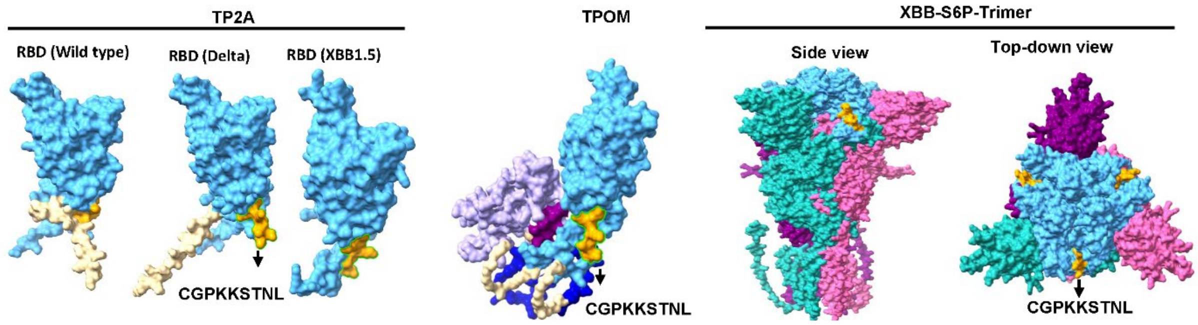


**Supplementary Figure 5:** The predicted position of the H-2D$^d$-restricted peptide epitope CGPKKSTNL (yellow) within RBD region (light blue) of the protein structure in mRNA vaccine candidates.
